# Epigenetic activation of HORMAD1 in basal-like breast cancer: role in Rucaparib sensitivity

**DOI:** 10.18632/oncotarget.25728

**Published:** 2018-07-10

**Authors:** Xian Wang, Ying Tan, Xixi Cao, Jin Ah Kim, Tianmeng Chen, Yiheng Hu, Matthew Wexler, Xiaosong Wang

**Affiliations:** ^1^ UPMC Hillman Cancer Center, University of Pittsburgh, Pittsburgh, PA 15232, USA; ^2^ Department of Pathology, University of Pittsburgh, PA 15232, USA; ^3^ Lester and Sue Smith Breast Center, Baylor College of Medicine, Houston, TX 77030, USA; ^4^ Dan L. Duncan Cancer Center, Baylor College of Medicine, Houston, TX 77030, USA

**Keywords:** HORMAD1, PARP inhibitor, basal-like breast cancer, xenograft

## Abstract

Basal-like breast cancer (BLBC) is an aggressive breast cancer subtype with features similar to the basal cells surrounding the mammary ducts. Treatment of patients with BLBC has been challenging due to the lack of well-defined molecular targets. Due to the clinical and pathological similarities of BLBC with BRCA-deficient breast cancers, the effectiveness of Poly (ADP-ribose) polymerase inhibitors (PARPi) has been tested in early phase clinical trials for patients with advanced BLBC, with limited clinical responses. Recently, it was reported that HORMAD1 overexpression sensitizes BLBC to HR-targeting agents by suppressing homologous recombination. Our independent analysis suggests that HORMAD1 is aberrantly overexpressed in about 80% of BLBC, and its expression in normal tissues is restricted to testis. Our experimental data suggests that HORMAD1 overexpression correlates with focal hypomethylation in BLBC. On the other hand, investigation of the Genomics of Drug Sensitivity in Cancer dataset revealed significantly reduced sensitivity of HORMAD1-overexpressing BLBC cell lines to Rucaparib, a commonly used PARPi. To further assess the role of HORMAD1 in PARPi sensitivity, we generated three HORMAD1-overexpressing xenograft models using the HORMAD1-low BLBC cell lines HCC1954, HCC1806, and BT20; we then subjected these xenograft models to Rucaparib treatment. Ectopic expression of HORMAD1 enhances tumor formations in two of these models, and significantly reduces sensitivity to Rucaparib in the HCC1954 model. Taken together, our data suggest that epigenetic activation of HORMAD1 by hypomethylation in BLBC may endow reduced sensitivity to Rucaparib treatment in some tumor models.

## INTRODUCTION

Basal-like breast cancer (BLBC) possesses a particularly aggressive clinical phenotype and a molecular subtype defined by an array of genes that are expressed by normal basal epithelial cells [1–3]. Most BLBCs do not express oestrogen receptor, progesterone receptor, or HER2 [4]. Although derived from luminal progenitors, BLBCs share a similar gene expression pattern with normal basal stem cells. This suggests that common epigenetic alterations underlie this cancer subtype. Despite many efforts to profile and sequence BLBC genomes, to date there are no defining genetic aberrations for this cancer subtype. Further, there is no generally accepted definition for basal-like breast cancer: Some groups have used immunohistochemical marker panels to define BLBC, while others have used microarray-based expression profiling to define BLBC. The later idea is becoming more dominant alongside the rapid development of sequencing technology and bioinformatics.

HORMAD1 encodes a HORMA domain-containing protein which binds to DNA double-strand breaks created during meiosis, promotes synapsis formation, and activates homologous recombination [5]. It was recently reported that HORMAD1 is overexpressed in triple-negative breast cancer (TNBC), and HORMAD1 expression sensitizes breast cancer cells to homologous repair (HR)-defect targeting agents by contributing to homologous recombination deficiency [6]. HORMAD1 is a testis germ cell protein that has been suggested to play roles in genomic instability in cancer [7]. However, the mechanism leading to HORMAD1 overexpression is unclear, and the role of HORAMD1 in PARP inhibitor sensitivity has not been assessed in preclinical xenograft models. As a parallel study, our group independently discovered that HORMAD1 is overexpressed in a subset of breast cancers, most of which belong to the basal-like subtype. In this study, we verify the frequency of HORMAD1 overexpression in BLBC, investigate the mechanism underlying ectopic overexpression of HORMAD1, and assess the effects of HORMAD1 overexpression on PARP inhibitor sensitivity in BLBC using preclinical xenograft mouse models.

## RESULTS

### HOMRAD1 overexpression is characteristic of basal-like breast cancer

**Figure 1:**
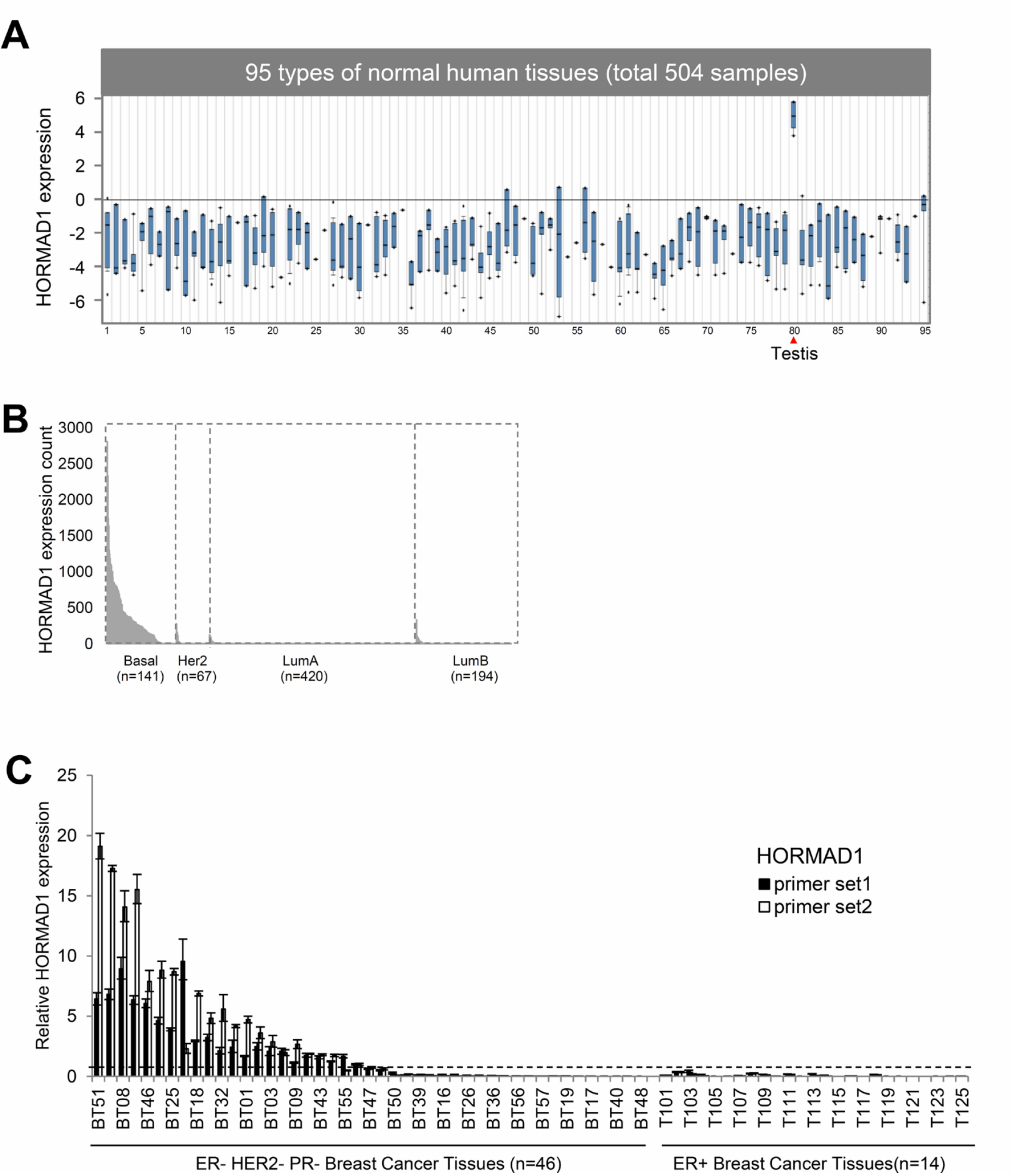
The expression of HORMAD1 in breast cancer tissues and normal human tissues (**A**) The expression of HORMAD1 in 504 samples from 95 types of normal human tissues are from the Human Body Index dataset (HBI, GSE7307) [8], and the data is visualized in Oncomine (http://www.oncomine.org) [34]. HORMAD1 expression is restricted to testis. ^*^Details of tissue identities can be found in Supplementary Table 3. (**B**) HORMAD1 expression in different breast cancer PAM50 subtypes based on TCGA RNAseq data. (**C**) HORMAD1 gene expression in 46 cases of TNBC and 25 ER+ breast cancer tissues was determined by qPCR using two independent HORMAD1 primer sets. The cut-off value for HOMRAD1 overexpression was defined as 0.57 (median + MAD (default constant = 1.4826)).

To examine HORMAD1 expression in normal human tissues, we analyzed Affymetrix U133 plus 2.0 microarray data for 34 normal tissue types from the GEO Human Body Index dataset (GSEA7307) [8]. This revealed that HORMAD1 expression in normal human tissues is strictly limited to testis and is repressed in most human somatic tissues (Figure 1A). To assess HORMAD1 expression in breast cancer subtypes, we analyzed RNAseq data for breast cancer from The Cancer Genome Atlas (TCGA) and associated HORMAD1 expression with receptor status, clinicopathological subtypes, and Prediction Analysis of Microarray 50 (PAM50) groups (Figure 1B, Supplementary Figure 1A). All samples are classified into two groups according to HORMAD1 expression level: overexpression (HORMAD1-high) and normal (HORMAD1-low). The overexpression cutoff was defined as median+1xMAD (see methods). Among breast cancer subtypes, HORMAD1 overexpression best associates with the BLBC subtype (83.6%), a stronger association than the TNBC subtype (69.6%) described previously [6] (Supplementary Figure 1B). Likewise, analysis of HORMAD1 overexpression frequency in the Metabric dataset [9, 10] suggests that HORMAD1 expression level negatively associates with ER status and is specifically elevated in BLBC (Supplementary Figure 1C). To verify the differential HORMAD1 expression in TNBC tissues compared to ER-positive tumors, we performed reverse transcription PCR (RT-PCR) using two primer sets specific for HORMAD1 in 14 ER positive and 46 triple-negative breast cancer tissues. Overexpression of HORMAD1 was detected in 52.2% of TNBC breast tumors but not in any of the ER-positive tumors (Figure 1C, Supplementary Table 2). This tumor panel does not have associated PAM50 data, and thus it cannot be used to evaluate differential HORMAD1 expression in different PAM50 subtypes.

To examine the signaling alterations characteristic of HORMAD1 overexpression, we analyzed the Reverse Phase Protein Array (RPPA) data for TCGA tumors [11]. The signaling proteins that are differentially expressed/phosphorylated in HORMAD1-high tumors compared to other BLBC tumors were identified using the R package Limma [12]. Several positive regulators of cell cycle and proliferation, such as S6 ribosomal protein and its upstream kinase P70S6K, GAB2, AKT, and cyclin B1, were up regulated in HORMAD1-high BLBC samples (Supplementary Figure 2). KU80, a protein known to function in DNA double strand break repair [13], was also up-regulated. These alternations implies the possible role of HORMAD in cell proliferation and DNA damage repair.

### Epigenetic activation of HORMAD1 overexpression in breast cancer tissues

**Figure 2:**
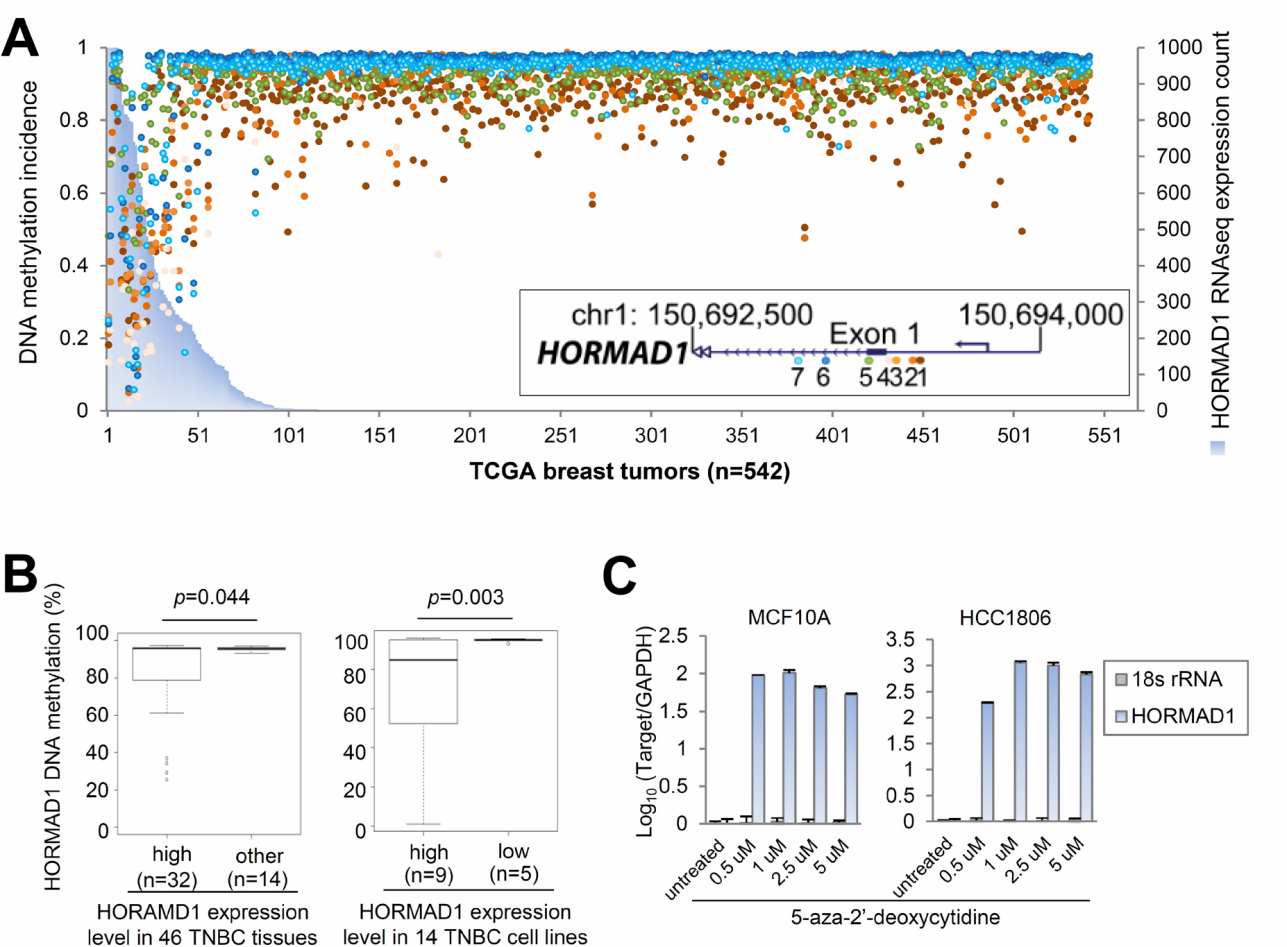
Ectopic expression of HORMAD1 in breast cancer is associated with DNA hypomethylation (**A**) Integrative analysis of TCGA RNAseq and epigenetic profiling data sets suggests that HORMAD1 overexpression associates with DNA hypomethylation around its promoter region in 542 TCGA breast tumor samples. Each colored dot represent a CpG site around the HORMAD1 promoter region in each TCGA breast tumor sample. The methylation percentage of each CpG site represents the fraction of methylation of each CpG site. (**B**) Pyrosequencing analysis was performed to investigate the HORMAD1 DNA methylation status in the same 46 cases of TNBC as well as 14 basal-like breast cancer cell lines. HORMAD1 DNA methylation percentage was plotted against HORMAD1 expression state. (**C**) Two HORMAD1-low breast cell lines, MCF10A and HCC1806 (a benign basal breast epithelial cell line and a BLBC line, respectively), were treated with 5′-Aza-2′-deoxycytidine, then HORMAD1 expression level was determined by qPCR using the qPCR primer set 1 (refer to methods or Supplementary Table 1 for details). The results after normalization (to housekeeping gene GAPDH) and log10 transformation suggest that HORMAD1 expression was substantially induced by 5′-Aza treatment at dosages as low as 0.5 uM.

To investigate the causes of HORMAD1 overexpression, we correlated HORMAD1 expression with copy number and DNA methylation at this locus using matched Affymetrix SNP 6.0 copy number data and Illumina Human DNA Methylation 450 data available from TCGA. Copy number data analysis suggests that HORMAD1 expression does not correlate with copy number (Pearson correlation: *R* = 0.214, Supplementary Figure 3A), but HORMAD1 overexpression significantly correlates with the hypomethylation status of its promoter region (Figure 2A, Supplementary Figure 3B). We therefore hypothesized that HORMAD1 overexpression may be driven by epigenetic activation.

To verify epigenetic activation of HORMAD1 overexpression, we investigated the methylation status of the HORMAD1 promoter region by Pyrosequencing in 45 TNBC tumors and 14 breast cancer cell lines. Our analysis revealed the presence of a CpG island at the HORMAD1 promoter region, which extends to most of the first exon (−127 bp∼+82 bp). We therefore examined the DNA methylation status of the HORMAD1 CpG island region by bisulfate pyrosequencing. HORMAD1 expression levels across breast cancer tissues were determined by qPCR (Figure 1C), and HORMAD1 expression levels across breast cancer cell lines was determined by Nanostring assay (Supplementary Figure 4). As expected, HORMAD1 expressions negatively correlate with methylation levels (Figure 2B, Supplementary Figures 5–6).

To further verify that HORMAD1 expression is regulated by DNA methylation, we examined whether HORMAD1 expression could be induced by the DNA demethylation agent 5-azacytidine (5-aza). We treated the MCF10A benign basal epithelial cell line and the HCC1806 basal breast cancer cell line, neither of which overexpress HORMAD1, with 5-aza. HORMAD1 demethylation and overexpression was substantially induced in both cell lines after 5-aza treatment, suggesting that regulation of DNA methylation could be one of the main mechanisms by which this gene is regulated (Supplementary Figure 5, Figure 2C). Taken together, our observations suggest that overexpression of HORMAD1 in BLBC could be regulated by epigenetic hypomethylation.

### PARP inhibitor sensitivity in HORMAD1-overexpressing breast cancer cell lines

**Figure 3:**
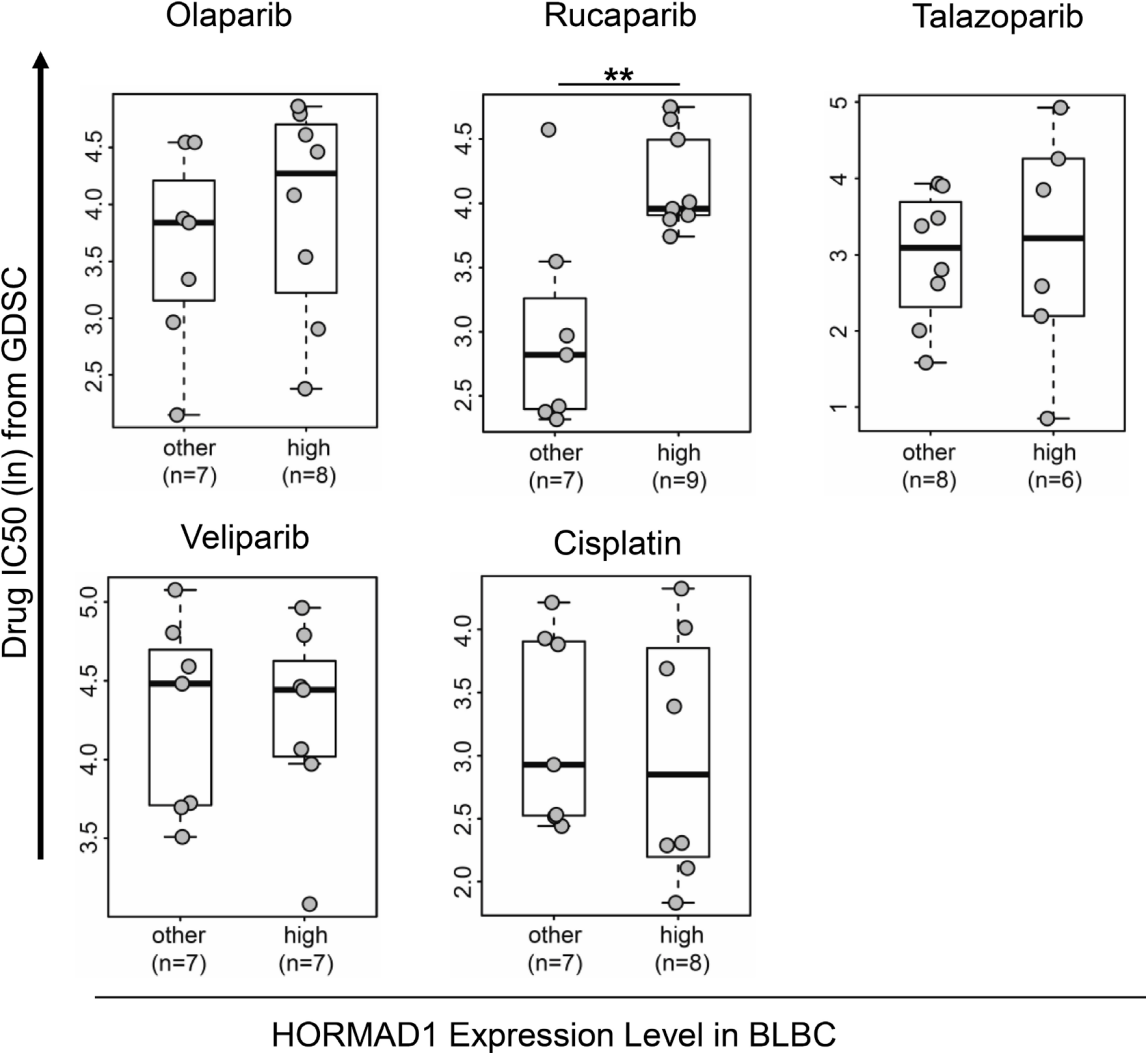
High HORMAD1 expression correlates with reduced PARP inhibitor sensitivity in basal-like breast cancer cell lines Drug response data and HORMAD1 expression data of cancer cell line panels were extracted from Genomics of Drug Sensitivity in Cancer (GDSC). IC50s of four PARP inhibitors (Olaparib, Rucaparib, Veliparib, and Talazoparib) and one commonly used DNA damaging agent (Cisplatin) from GDSC are plotted against HORMAD1 expression status in BLBC cell lines without known BRCA mutations [25]. Statistical significance was determined by a two-tailed Student’s *t*-test. ^*^P < 0.05.

PARP inhibitors (PARPi) have shown promising clinical effect on BRCA-mutant ovarian and breast cancer [14]. And most recently FDA has approved the first PARP inhibitor for breast cancer which has demonstrated favorable response rate in HER2-negative, metastatic breast cancer with germline BRCA mutation (59.9%). Since HORMAD1 is known to function in homologous recombination during meiosis, we speculated that HORMAD1 overexpression may affect PARP inhibitor sensitivity in BLBC. To test this, we analyzed the sensitivity of HORMAD1-high and HORMAD1-low cell lines to PARP inhibitors using the Genomics of Drug Sensitivity in Cancer (GDSC) dataset [15]. The Genomics of Drug Sensitivity in Cancer project is a collective dataset that combines genomics data with drug activity data for 265 chemical compounds in 1074 cancer cell lines from distinct tumor entities. The expression levels of HORMAD1 in the GDSC profiled cell lines were defined based on the matched gene expression data from GDSC. In addition to PARP inhibitors, we also assessed the correlation of the sensitivity of another available DNA damaging agent, Cisplatin, with HORMAD1 overexpression. Consistent with a previous report [6], the data from GDSC suggested that HORMAD1-high BLBC tumors tend to be more sensitive to Cisplatin treatment than the rest BLBC tumors (Figure 3). We then analyzed the four PARP inhibitors included in this dataset, Olaparib, Rucaparib, Talazoparib, and Veliparib. Interestingly, most of the PARP inhibitors tested trend toward lower efficacy in HORMAD1-high cell lines, and we found that HORMAD1-high cell lines showed significantly reduced sensitivity to Rucaparib compared to other cell lines.

### The effect of HORMAD1 overexpression on Rucaparib sensitivity *in vivo*

**Figure 4:**
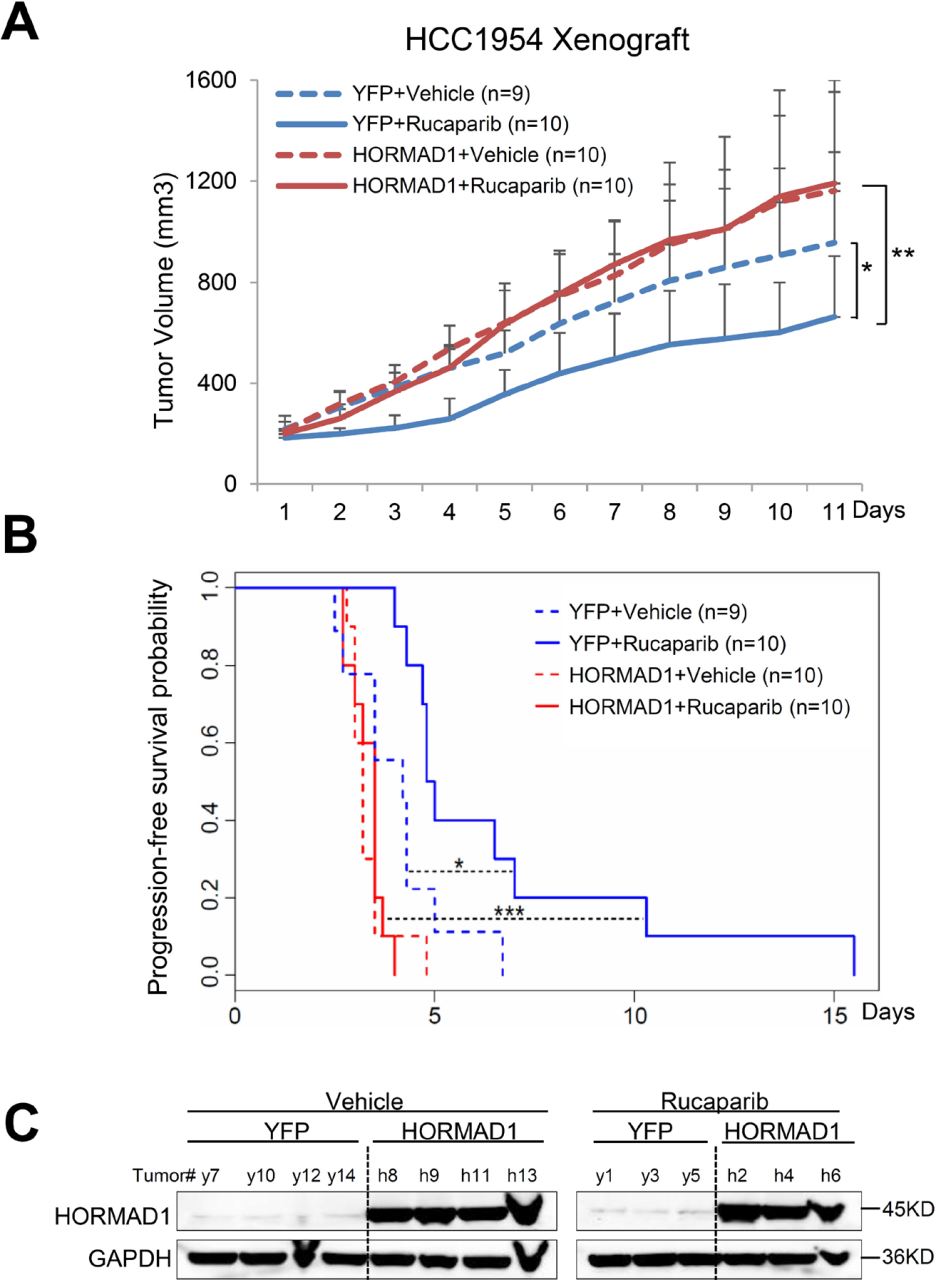
HORMAD1 increases tumor growth rate and endows reduced sensitivity to Rucaparib in the HCC1954 xenograft model (**A**) HORMAD1 overexpression in the engineered HCC1954 xenograft tumors accelerates tumor growth independent of Rucaparib treatment. Tumor growth was tracked daily after inoculation. Average tumor volume of each group was used to plot the growth curve. Error bars represent the standard deviation of tumor volume in each group. The difference between groups was calculated by ANOVA. **(B**) Progression-free survival of HORMAD1-overexpressing tumors was reduced compared to YFP control tumors with or without Rucaparib treatment. Tumor Progression-free period was defined by the time it takes for the tumor volume to double after establishment of the tumor. Kaplan–Meier Tumor Progression-Free Survival (PFS) analysis was performed in R survival package. (**C**) HORMAD1 expression level in the engineered HCC1954 xenografts was verified by Western blot. Three to four tumors were randomly selected from each treatment group then subjected to western blot assay.

**Figure 5:**
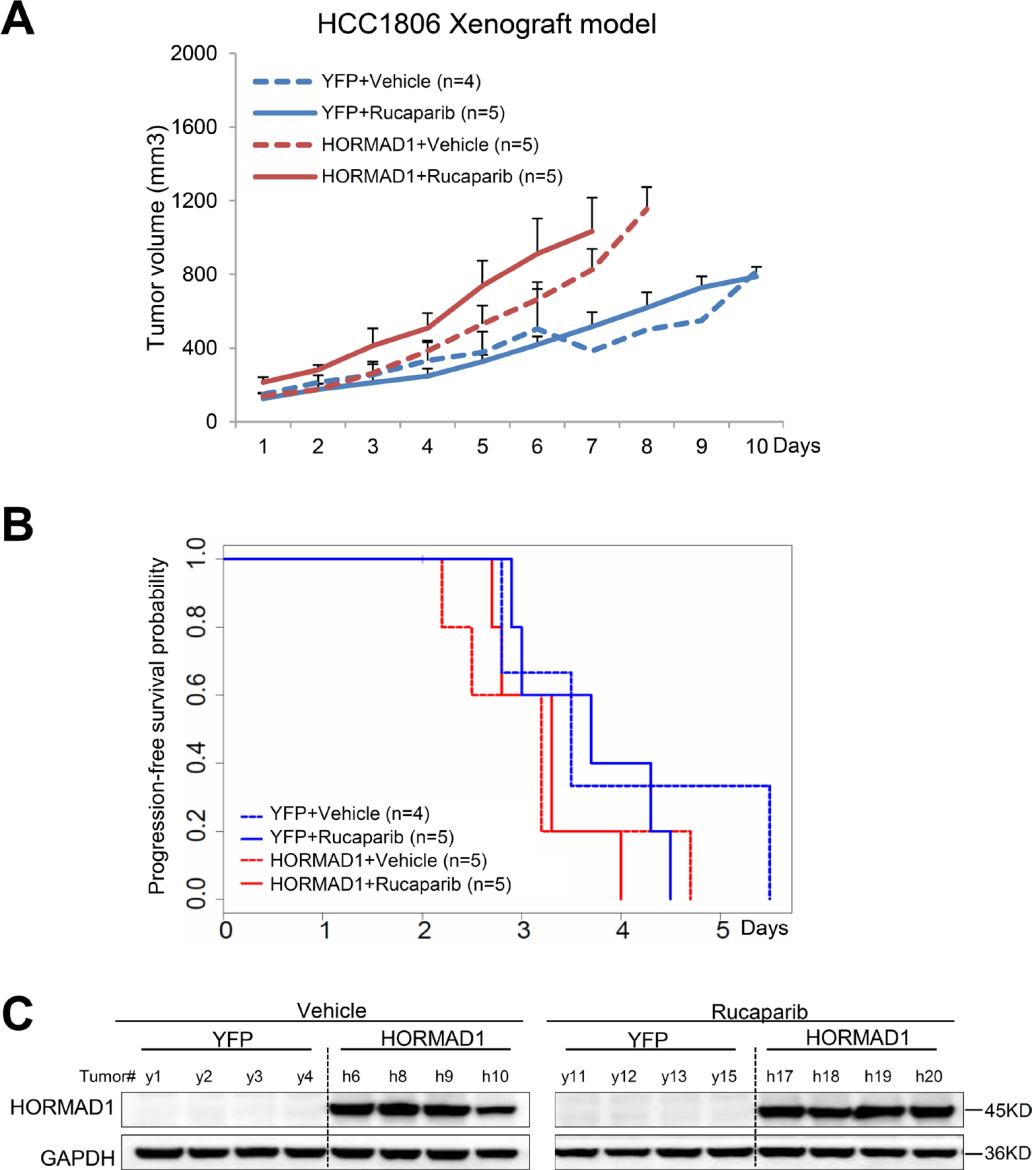
The HCC1806 xenograft model does not respond to Rucaparib, regardless of HORMAD1 overexpression, and overexpression of HORMAD1 moderately increases tumor growth independent of Rucaparib treatment (**A**) The growth curve YFP/HORMAD1 engineered HCC1806 xenograft tumors with or without Rucaparib treatment. Tumor growth was tracked daily after inoculation. Average tumor volume of each group was used to plot the growth curve. Error bars represent the standard deviation of tumor volume in each group. The difference between groups was calculated by ANOVA. (**B**) Progression-free survival of HORMAD1-overexpressing tumors compared to YFP control tumors with or without Rucaparib treatment. Tumor Progression-free period was defined by the time it takes for the tumor volume to double after establishment of the tumor. (**C**) HORMAD1 overexpression in the engineered HCC1806 tumor tissue was validated by Western blot. Four tumors were randomly selected from each treatment group then subjected to western blot assay.

**Figure 6:**
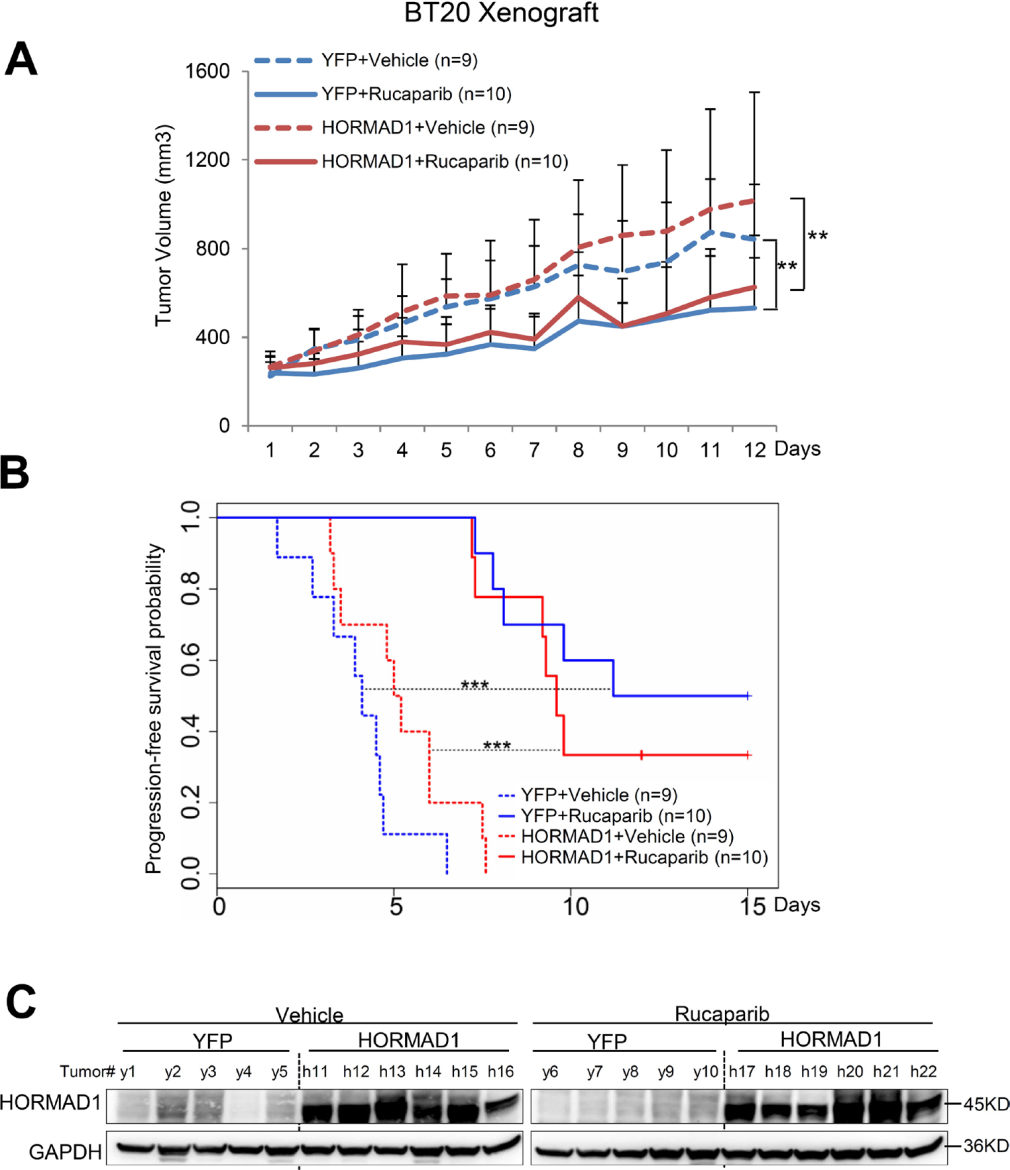
HORMAD1 overexpression does not change tumor growth rate or the effect of Rucaparib in the BT20 xenograft model (**A**) Growth of BT20 xenograft tumors was significantly inhibited by Rucaparib treatment. Overexpression of HORMAD1 did not affect tumor growth rates. Tumor growth was tracked daily after inoculation. Average tumor volume of each group was used to plot the growth curve. Error bars represent the standard deviation of tumor volume in each group. The difference between groups was calculated by ANOVA. (**B**) Progression-free survival of HORMAD1-overexpressing tumors compared to YFP control tumors with or without Rucaparib treatment. Tumor Progression-free period was defined by the time it takes for the tumor volume to double after establishment of the tumor. (**C**) HORMAD1 overexpression in engineered BT20 xenograft tumor tissue was validated by Western blot. Five to six tumors were randomly selected from each treatment group then subjected to western blot assay.

To further test the association of HORMAD1 overexpression with tumor response to Rucaparib treatment *in vivo*, we generated several xenograft mouse models using BLBC cell lines engineered to ectopically overexpress HORMAD1 and assessed the tumors’ responses to Rucaparib treatment. We selected three HORMAD1-low BLBC cell lines, HCC1954, HCC1806, and BT20, then engineered these cell lines to ectopically express either HORMAD1 or yellow fluorescent protein (YFP) as a control. The engineered lines were inoculated into fat pads of female athymic nude mice. Upon tumor establishment, mice bearing either HORMAD1-overexpressing tumors or YFP-expressing tumors were randomized and treated with Rucaparib (10 mg/kg) or the vehicle phosphate-buffered saline (PBS). HORMAD1 protein levels in the xenograft tumors were verified by western blots (Figures 4–6).

Even though all of these cell line models are characterized as BLBC [16, 17], we found that they behave very differently from each other when subjected to Rucaparib treatment and HORMAD1 overexpression (Supplementary Figure 7). We found that HORMAD1 overexpression significantly increased tumor growth in HCC1954 xenograft tumors compared to YFP-expressing controls (Figure 4). On the other hand, HORMAD1 overexpression only lead to moderately increased tumor growth in the HCC1806 xenograft model (Figure 5), while BT20 xenograft growth is not affected by HORMAD1 protein level at all (Figure 6). While subjected to Rucaparib treatment, overexpression of HORMAD1 in the HCC1954 model reduced tumor sensitivity to Rucaparib treatment (Figure 4), which is consistent with our analyses of cell line data (Figure 3). However, HCC1806 xenografts did not exhibit sensitivity to Rucaparib in either YFP or HORMAD1-overexpressing tumors (Figure 5), which suggests that HORMAD1-independent cellular processes may also confer resistance to Rucaparib treatment. It is also noteworthy that HCC1806 was derived from primary acantholytic squamous cell carcinoma of the breast, whereas the other two models were derived from breast adenocarcinoma. This suggests that the genetic nature of the tumor may also impact how the tumor responds to Rucaparib treatment.

In the BT20 model which bears a BRCA2 mutation [18], Rucaparib treatment decreased tumor growth as expected, but HORMAD1 overexpression did not affect baseline tumor growth or response to Rucaparib. This suggests that the reduced sensitivity to Rucaparib endowed by HORMAD1 in BLBC may rely on the intact BRCA function. Further studies will be needed to define the precise mechanism(s) which govern the effect of HORMAD1 activity on sensitivity to PARP inhibitor treatment.

## DISCUSSION

The characteristics of BLBC have been intensively studied in the past decade. However, due to the lack of a universally-accepted genetic signature, the definition of basal-like breast cancer remains debatable. To date, BLBC has been characterized by a lack of estrogen receptor, lack of *ERBB2* amplification, and high mitotic rate [1–3]. In this study, we show that HORMAD1 is preferentially overexpressed in the BLBC subtype of breast cancer based on analysis of the TCGA and Metabric datasets. HORAMD1 is testis-specific germ cell protein that is known to be reactivated in some cancer types, where it has been proposed to play roles in genomic instability and drug resistance [7]. Because HORMAD1 overexpression is preferentially present in BLBC, BLBC could be characterized in part by HORMAD1 overexpression. Meta-analysis of genomic data and experimental data suggest that HORMAD1 overexpression is caused by hypomethylation of its promoter region.

BLBC often presents with an aggressive clinical phenotype, including high rate of recurrence, distant metastasis, and short patient survival period [19–21]. Effective targeted therapies are desperately needed to treat this disease; however, to date, no efficacious drug has been developed. This is due in large part to the lack of a viable molecular target in BLBC. PARP inhibitors have been proposed as one strategy to treat patients with BLBC. Although some promising data supports the use of PARP inhibitors in BLBC treatment [22], and PARP inhibitors have been subjected to clinical trials to treat metastatic breast cancer [23], the use of these drugs for consistent clinical benefit will be more efficient with viable predictive biomarkers that can be used in addition to BRCA mutation. A recently published paper suggested that BRCA1/2 mutation status is not an accurate prediction marker for PARP inhibitor response, further underscoring the need for additional complementary biomarkers [18].

Our group and others have discovered the association of HORMAD1 overexpression with the response to certain PARP inhibitor treatment; however, some disparities exist in these observations. For example, a recent report suggests that HORMAD1 overexpression endows sensitivity to Olaparib in TNBC tumors [6]. Considering that HORMAD1 is overexpressed in a vast majority of TNBC tumors, if high HORMAD1 expression endows sensitivity of TNBC tumors to Olaparib treatment, a large proportion of TNBC tumors would be expected to be sensitive. Our analysis of GDSC data showed that cell lines overexpressing HORMAD1 are significantly less sensitive to the PARP inhibitor Rucaparib, and cell lines which overexpressed HORMAD1 trended towards decreased sensitivity to other PARP inhibitors, including Olaparib and Veliparib. These discrepancies may be explained in part by the different cell lines included in the respective studies: Among the 18 BLBC cell lines included in GDSC and the nine BLBC cell lines included in Watkins *et al.*’s study, only five cell line were used in both studies. Additionally, the classification of HORMAD1-high or –low BLBC between Watkins’s study and GDSC datasets are based on different batches of measurements by different microarray platforms, which resulted in one of the HORMAD1-high cell lines in Watkins’s study was otherwise in GDSC. The lack of comprehensiveness in cell line panel and consistency in expression measurements could contribute to the different observations between these datasets. Future studies which include more BLBC cell lines and xenograft tumors will help to clarify these findings.

It is worth mentioning that in the *in vitro* studies, cells can only be exposed to PARP inhibitors for a short period of time, while a longer drug exposures are often required to observe significant effects of PARP inhibitors. We therefore used *in vivo* preclinical models to investigate the correlation between PARP inhibitor sensitivity and HORMAD1 overexpression based on longer term exposure to Rucaparib treatment. In addition, we have chosen to apply HORMAD1 overexpression model instead of genetic inhibition, as the acute loss of HR repair genes such as BRCA1 have been shown to be toxic to cancer cells [24, 25]. Thus, even if Rucaparib-sensitizing effects are observed following HORMAD1 inhibition, we cannot rule out the cytotoxicity effect caused by acute loss of HORMAD1 expression. Our results showed that HORMAD1 overexpression endowed reduced sensitivity to Rucaparib treatment in the HCC1954 xenograft model, which was not observed in the Rucaparib-sensitive BT20 xenograft model, or the HCC1806 xenograft model which was not sensitive to Rucaparib treatment independent of HORMAD1 expression. This demonstrates that, at least in some biological contexts, HORMAD1 overexpression is sufficient to render reduced sensitivity to Rucaparib treatment. It is worth mentioning that even though all these models are classified as BLBC, the genetic background of HCC1954, HCC1806, and BT20 are heterogeneous: For instance, the BT-20 cell line bears a BRCA2 mutation [18], which may endow sensitivity to Rucaparib treatment independent of HORMAD1 overexpression, whereas the other two cell lines do not harbor BRCA mutations. PARPi have been used in the treatment of BRCA-mutant breast and ovarian cancers [26, 27]. This suggests that aberrant HORMAD1 expression may confer reduced sensitivity to PARPi treatment in a manner that is dependent on the genetic background of the cells. In addition, the HCC1806 cell line is an acantholytic squamous cancer, not breast adenocarcinoma, which may explain its resistance to rucaparib treatment irrespective of HORMAD1 overexpression.

Together, the different genetic background of the cell line models used in our *in vivo* studies might explain why the HCC1954 and BT-20 xenograft models are sensitive to Rucaparib treatment, and why only HCC1954 demonstrate reduced sensitivity to Rucaparib with ectopic HORMAD1 overexpression. Further studies will be needed to identify the additional mechanisms which modulate PARP sensitivity in the presence of HORMAD1 overexpression, which could be used alongside HORMAD1 as predictive markers for PARP inhibitor treatment. A comprehensive understanding of the factors, including HORMAD1, which influence tumors’ responses to PARP inhibitor treatment will allow clinicians to design treatment strategies with the best chance of success in combating BLBC.

## MATERIALS AND METHODS

### Cell line and tissue collections

HCC1143, HCC1937, HCC1954, sum102PT, HCC1806, and BT20 breast cancer cell lines were obtained from American Type Culture Collection (ATCC). All breast tumor patient tissues were obtained from the Tumor Bank of Lester and Sue Smith Breast Center at Baylor College of Medicine. 293FT cells used for lentivirus packaging were purchased from Invitrogen. HCC1143, HCC1937, HCC1954, and HCC1806 cells were cultured in RPMI 1640 (Cellgro) with 10% fetal bovine serum (Thermo Fisher Scientific). BT20 cell was cultured in DMEM (Thermo Fisher Scientific) with 10% fetal bovine serum. Sum102PT was cultured in Ham’s F-12 supplemented with 5 ug/ml insulin, 1 ug/ml Hydrocortisone, 10 ng/ml EGF, 5 mM Ethanolamine, 10 mM HEPES, 5 ug/ml Transferrin, 10 nM Triiodothyronine, 50 nM Sodium Selenite, 1 g/L Bovine Serum Albumin.

### 5-aza-2′dc drug treatment and qPCR

5-azadC was purchased from Sigma. 5-azadC was dissolved in 1× phosphate-buffered saline (PBS, PH 7.0). In methylation inhibition experiments, cells were treated with 0.5–5 uM for 24 hours. Cells were then harvested and expression levels of HORMAD1 were analyzed by qPCR. Complementary DNA was generated from 1 ug of total RNA using the Transcriptor First Strand cDNA synthesis Kit (Roche) in the presence of both oligo (dT) and random primers. RT-PCR of HORMAD1 was performed with Platinum Taq High Fidelity (Invitrogen) and primers (Primer set 1: Forward primer: GCCCAGTTGCAGAGGACTC, Reverse primer: TCTTGTTCCATAAGCGCATTCT; Primer set 2: Forward primer: TGGCAAATGGAAATCAACCAGT; Reverse primer: TGCAAGCCTGCAGAACAAAA). Detail about primers can be found in Supplementary Table 1. A designated clean room was used for setting up PCR reactions to avoid potential contamination. In addition, a special set of pipettes and wipes with aerosol filters was used to set up the PCR reaction. cDNA samples were subjected to 35 PCR cycles of 94° C for 30 sec, 56° C for 30 sec, and 68° C for 2 min. To quantify the RT-PCR results, band intensities were quantified using ImageJ software (National Institutes of Health) and normalized to respective GAPDH controls.

### Pyrosequencing methylation analysis

DNA methylation of HORMAD1 was determined by bisulfite pyrosequencing. Bisulfite conversion of genomic DNA (1 ug each) of 45 Triple Negative tissues was performed using the EZ DNA methylation kit (Zymo D5001) according the manufacturer’s instructions. A CpG island around the transcription start site was amplified from bisulfite-converted DNA (Forward primer: GATTAGGGGTTAAAAAGTTATT; reverse primer: Biotin_CCATCTCAAAAACCTCTATTA). The methylation status of 11 CpG sites within this CpG island were examined in a Qiagen pyrosequencing machine at MD Anderson DNA Methylation Analysis Core (Sequencing primer 1: GGGGTTAAAAAGTTATTG; Sequencing primer 2: GGTGATYGTTGAAGGAAAG). The relative expression level of HORMAD1 in each of the tissues was then determined by qPCR (Forward primer: GCCCAGTTGCAGAGGACTC, Reverse primer: TCTTGTTCCATAAGCGCATTCT). Detail of primers can be find in Supplementary Table 1.

### Nanostring assay

The code set (Version.2) for *HORMAD1* (Probe NSID: NM_032132.3:459, NM_032132.4:901) was designed by Nanostring Technologies based on the gene sequence. Expression of this gene was quantified from 500 ng total RNA using the Nanostring Counter Assay System following the manufacturer’s instructions. Raw counts were normalized to the mRNA levels of the house-keeping genes *TFRC*, *TBP*, and *PUM1*.

### Bioinformatics analysis of gene expression

Affymetrix U133 plus 2.0 microarray data for 34 normal tissue types were obtained from the Human Body Index dataset (HBI, GSE7307). Gene expression values were extracted with the MAS5 algorithm and were scaled to a reference sample using a house-keeping gene probe set provided by Affymetrix as previously described [28]. RNA-seq based expression data, Affymetrix SNP 6.0 array based CNV data, and Illumina Infinium Human DNA Methylation 450 data were obtained from the level 3 data in TCGA portal (https://portal.gdc.cancer.gov/). In addition, we also analyzed the Molecular Taxonomy of Breast Cancer International Consortium (Metabric dataset, Illumina HT-12 V3) [9]. HORMAD1 overexpression is defined based on the cutoff of median + 1 × MAD (median absolute deviation) as in our previous study [29]. MAD is calculated using R with the default constant. PAM50-based clinical subtypes of breast cancer for TCGA samples were derived from the UCSC Cancer Genome Browser (https://genome-cancer.ucsc.edu/) [11, 30]. HORMAD1 copy number data was summarized from the segmented level 3 data, and the genomic instability index was calculated based on copy number data as previously described [31]. The TCGA methylome and RNAseq expression data from breast cancers were analyzed and visualized by MEXPRESS [32].

### Bioinformatics analysis of GDSC dataset

IC50 values of 1074 human pan-cancer cell lines against 265 compounds were obtained from Genomics of Drug Sensitivity in Cancer (GDSC)15 website (https://www.cancerrxgene.org/downloads). The included PARP inhibitors are Olaparib (Drug ID: 1017 & 1495), Veliparib (Drug ID: 1018), Rucaparib (Drug ID: 1175), and Talazoparib (Drug ID: 1259). Gene expression data of human cancer cell lines were downloaded from the GDSC web portal, from which HORMAD1 expression data were extracted. Cell lines with both gene expression data and drug sensitivity data were selected to study the relationship between HORMAD1 overexpression and sensitivity to PARP inhibitors. The threshold for HORMAD1 overexpression was calculated by median + MAD (default constant = 1.4826). MAD is calculated by mad () function in R with the default constant. For pan-cancer analysis, the threshold was set up based on all cell lines in the dataset. For the analysis of all breast cancer cell lines or basal-like breast cancer cell lines, the threshold was set up based on all breast cancer cell lines. After that, cell lines were divided into two groups based on the status of HORMAD1 expression (high or other). IC50 value was plotted against HORMAD1-expression status. *T*-test was adopted to evaluate the differences of IC50 values between two groups. *P*-value was calculated on log-transformed values. *p* < 0.05 was regarded as statistical significant. Boxplots represent 1st, 2nd, and 3rd quantiles.

### Bioinformatics analysis of TCGA-RPPA data

To assess the cell signaling alterations characteristics of HORMAD1 overexpression in BLBC tumors, we downloaded the RBN (replicate-base normalization) normalized reverse phase protein array (RPPA) data for 747 TCGA breast cancer tumors from Xena Functional Genomics Explorer (https://xenabrowser.net) [33]. Using the available RPPA data for 121 TCGA BLBC samples, we applied the R package Limma (Linear models for microarray and RNA-seq analysis) [12] to identify proteins differentially expressing in HORMAD1-high tumors vs the remaining BLBC tumors. The threshold for HORMAD1-high was calculated by median+MAD using RNA-seq expression data, as described above. The significantly altered molecules were visualized in heat-map and sorted by limma *p*-value.

### Engineering HORMAD1 ectopic overexpression cell line models

Human HORMAD1 sequence verified cDNA was obtained from Dharmacon (MHS1010-9204021). The cDNAs for HORMAD1 and YFP control were then cloned into the pcDNA3 vector (Invitrogen). These lentiviral constructs verified by sequencing before being infected into selected cell lines using the ViraPower^™^ Lentiviral Support Kit (Invitrogen). Cells with high GFP reporter expression were selected using flow cytometry.

### *In vivo* xenograft experiments and reagents

All animal studies were performed in accordance with protocols approved by BCM Institutional Animal Care and Use Committee. 1 × 10^7^ transduced HCC1954, HCC1806, or BT20 cell lines, with either HORMAD1 overexpression or YFP vectors, were re-suspended in 20% Matrigel (VWR) solution. Cells were injected bilaterally in 4–6-week-old female athymic nude mice (Harlan Sprague-Dawley). For *in vivo* therapeutic efficacy evaluation, the mice bearing engrafted tumor were randomized into four treatment arms: YFP/PARPi+, YFP/PARPi-, HORMAD1 /PARPi+, and HORMAD1/PARPi-. Rucaparib used in animal studies was obtained from Selleck Chemicals. 10 mg/kg Rucaparib or vehicle was administered to mice by oral gavage using a blunt needle. The growth of xenograft tumors was monitored daily and tumor volume was measured using the formula 1/2(length × width^2^). Mice were terminated and tumors were collected when tumors reach 1500 mm^3^ or at the designated endpoint of the experiment.

### Western blots and antibodies

Xenograft tumor tissues were extracted in RIPA lysis buffer with complete protease inhibitor cocktail supplements (Roche). Protein samples were separated in SDS-PAGE gel and then transferred to 0.2 uM nitrocellulose membrane. The primary HORMAD1 antibody (SIGMA HPA037850) was diluted by 1:1000 ratio in 5% BSA-PBST buffer for blotting. The primary GAPDH antibody was purchase from Santa Cruz (#4970) and works under 1:2000 dilution. The western blots were visualized in Bio-Rad ChemiDoc imaging system and processed by ImageJ software (National Institutes of Health).

### Statistical analysis

The *in vitro* experiment data were analyzed by unpaired Student’s *t*-tests, and all data are shown as mean ± standard deviation. Analysis of *in vivo* tumor growth curves was carried out using ANOVA. Kaplan–Meier survival analysis was carried out using the R survival package to estimate the function of Rucaparib on progression-free survival in BLBC with different HORMAD1 levels. Tumors were considered “progression-free” until the measured tumor volume was more than double the tumor volume at the beginning of the experiment, at which point the tumors were deemed to have “progressed.” Comparisons between survival curves were carried out by generalized Wilcoxon test.

## SUPPLEMENTARY MATERIALS FIGURES AND TABLES








