# Supplementary material for: Epigenetic activation of HORMAD1 in basal-like breast cancer: role in Rucaparib sensitivity
Source: Oncotarget. 2018 Jul 10;9(53):30115–27. doi: 10.18632/oncotarget.25728 (PMC6059019; doi:10.18632/oncotarget.25728)
Supplement: Supplementary file 1 [file oncotarget-09-30115-s001.pdf]

Epigenetic activation of HORMAD1 in basal-like breast cancer: role in Rucaparib sensitivity

SUPPLEMENTARY MATERIALS

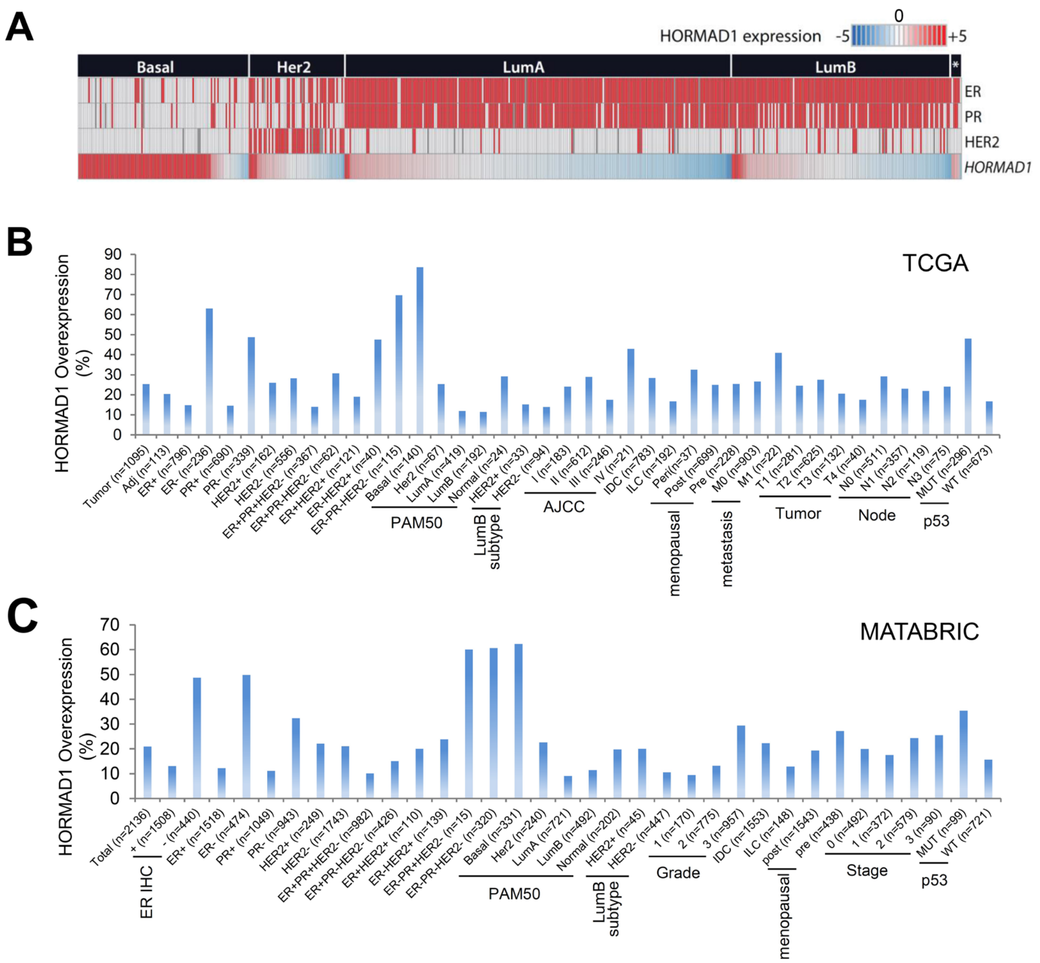

Supplementary Figure 1: HORMAD1 RNAseq expression in different breast cancer subtypes. (**A**) Relative expression levels of HORMAD1, ER, PR, and Her2 in different PAM50 breast cancer subtypes are shown as a heat-map. HORMAD1 expression is enriched in Basal-like breast cancer. (**B**) The frequency of HORMAD1 overexpression (determined by RNAseq data) in different breast cancer subtypes, as well as clinical associations from the TCGA dataset. (**C**) The frequency of HORMAD1 overexpression in different breast cancer subtypes as well as clinical associations from the Metabric dataset. In both (B and C), HORMAD1 is overexpressed most frequently in BLBC and TNBC subtypes.

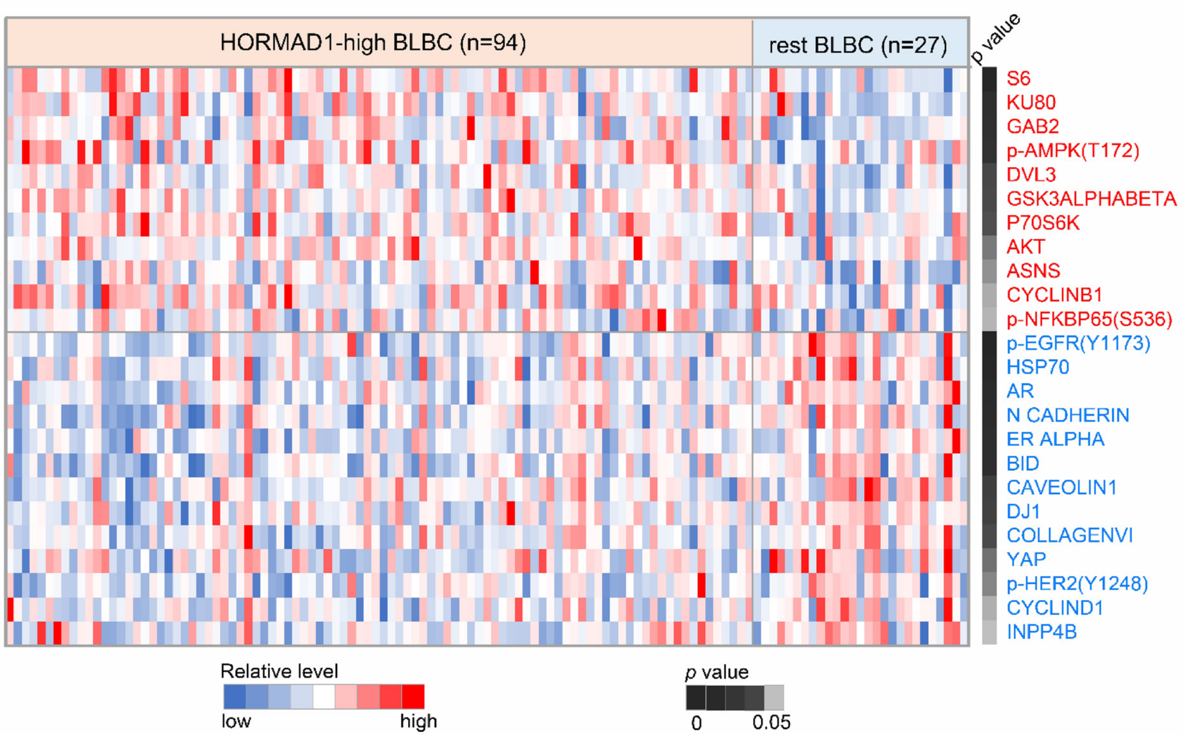

Supplementary Figure 2: Linear Models for Microarray and RNA-seq Analysis (Limma) of TCGA RPPA data identifies the proteins characteristic of HORMAD1 overexpression in BLBC tumors. 121 TCGA BLBC samples with available RPPA data were classified into two groups: HORMAD1-high ($n = 94$) and the rest ($n = 27$) according to HORMAD1 expression levels (RNA-seq data). Differentially expressed and/or phosphorylated signaling proteins were identified by Limma statistics comparing the HORMAD1-high with rest of the BLBC tumors, and were plotted in the heatmap sorted by their Limma *p*-value.

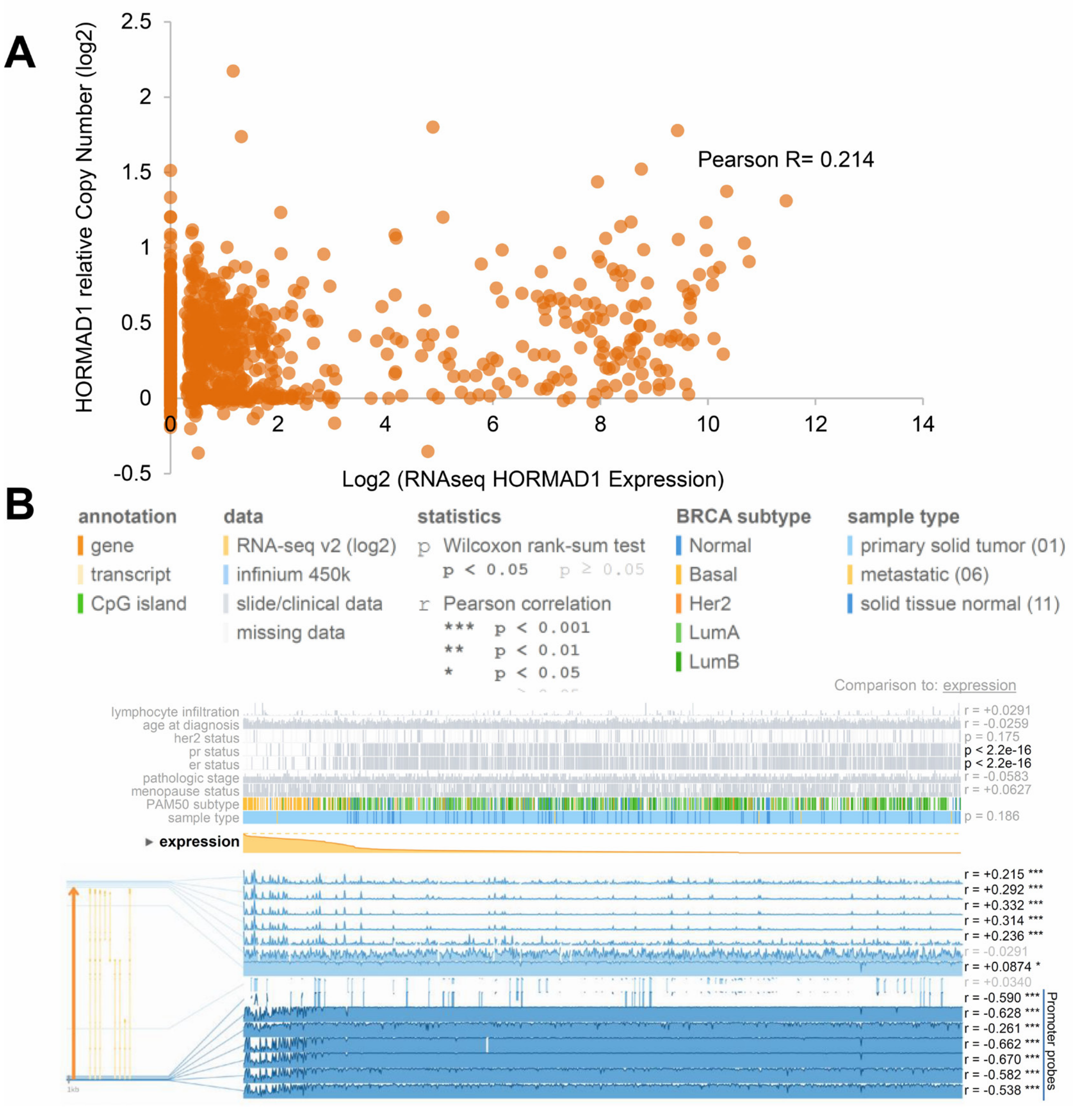

Supplementary Figure 3: Relationship between HORMAD1 expression level and multiple factors. (**A**) HORMAD1 expression does not correlate with HORMAD1 copy number. HORMAD1 copy number and RNAseq expression data were extracted from 1097 TCGA breast cancer samples and plotted against each other. (**B**) HORMAD1 expression (yellow graph), methylation for each probe location (blue graphs), and clinical data of 871 TCGA breast cancer samples were plotted against each other using the MEXPRESS web tool. The samples are ordered by HORMAD1 expression level, revealing increased HORMAD1 expression correlates with decreased methylation. The number on the far right indicates the significance of the relationship (correlation coefficient or *p*-value) between each row of data (clinical, expression, or methylation) and HORMAD1 expression. The correlations between HORAMD1 expression and PAM50 subtype, as well as receptor status, suggest that HORAMD1 could be a characterizing factor for BLBC.

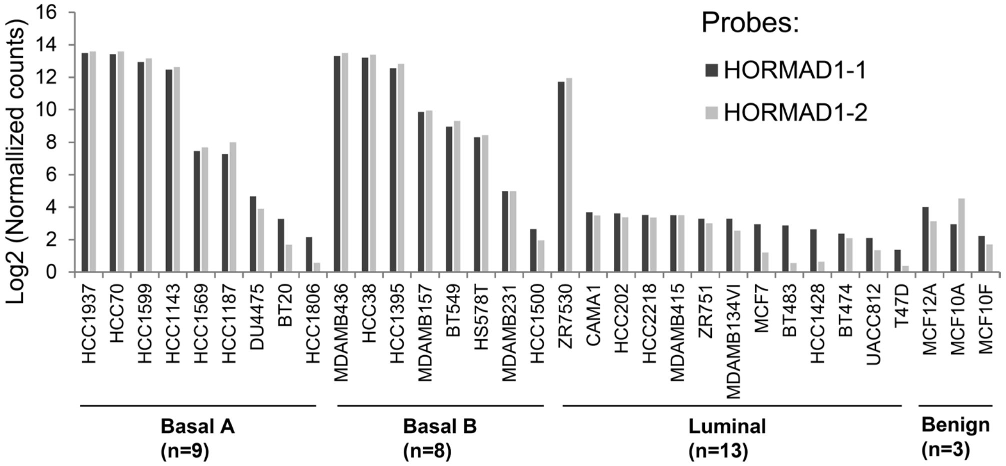

Supplementary Figure 4: HORMAD1 expression level in multiple breast cell lines. Expression of HORMAD1 in multiple breast cancer cell lines (*n* = 30) and noncancerous breast epithelial cells (*n* = 3) was determined by Nanostring analysis applying two independent probes: HORMAD1-1 and HORMAD1-2. Breast cancer cell lines were classified into three sub-groups: basal A, basal B, and luminal. HORMAD1 expression is enriched in cell lines of both basal-like breast cancer subtypes compared to luminal breast cancer cell lines.

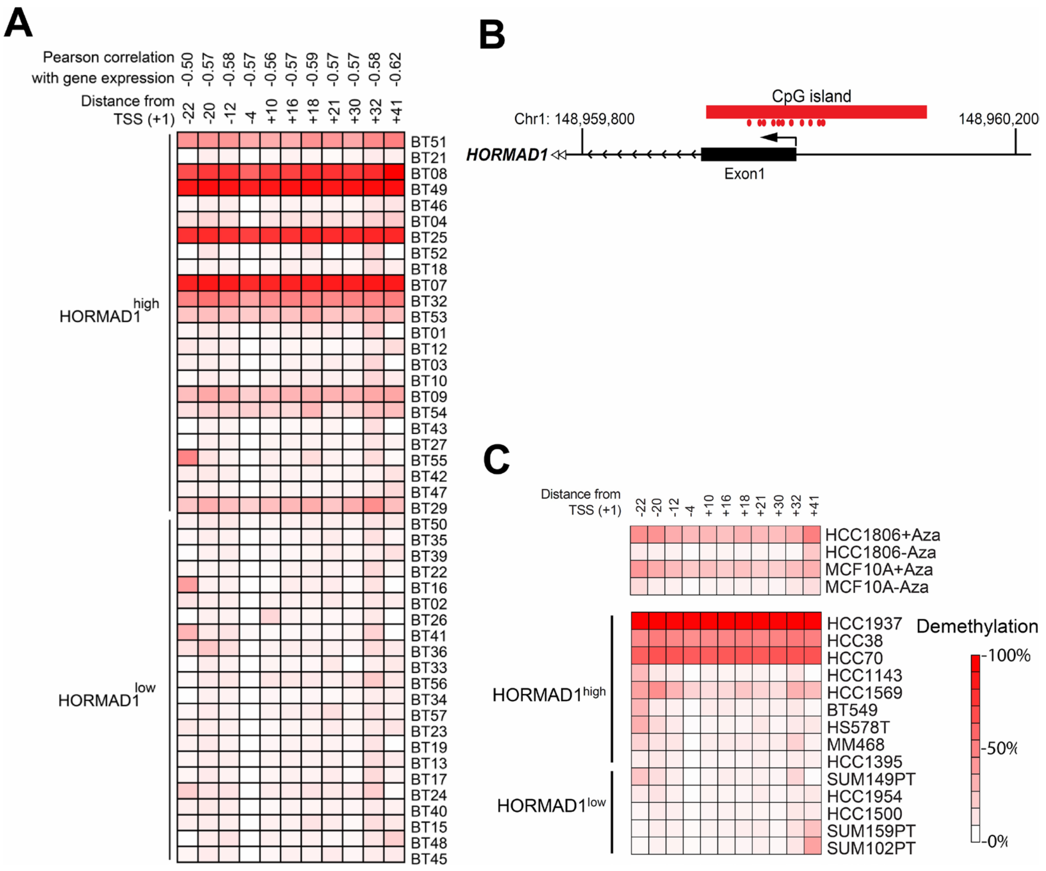

Supplementary Figure 5: Methylation status around the transcription start site (TSS) of the HORMAD1 gene in TNBC tissues (*n* = 46) and cell lines (*n* = 14) as determined by pyrosequencing analysis. (**A**) HORMAD1 expression level correlates with hypomethylation of the CpG sites in the region of the HORMAD1 gene containing the TSS and the first exon in 46 TNBC tissues. (**B**) A schematic figure to present the positions of the CpG methylation sites around the TSS. (**C**) HORMAD1 expression level correlates with demethylation percentage of the CpG sites around the TSS in TNBC cell lines. 5'-Aza treatment in two TNBC lines, HCC1806 and MCF10A, increases demethylation.

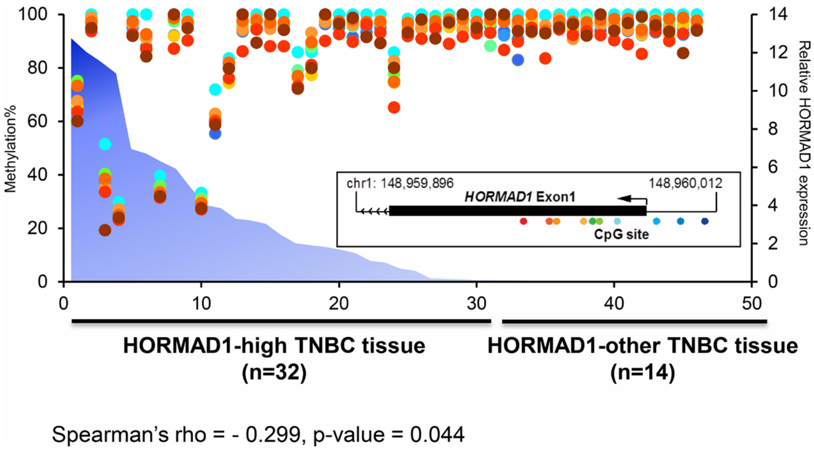

Supplementary Figure 6: Expression of HORMAD1 in TNBC tissues (*n* = 46) significantly correlates with hypomethylation status of the CpG sites in the region of the HORMAD1 gene containing the promoter and the first exon. DNA hypomethylation status around the HORMAD1 promoter region in 46 TNBC breast tumor samples was determined by pyrosequencing analysis. Each colored dot represent a CpG site around the HORMAD1 promoter region in each breast tumor sample. The methylation percentage of each CpG was plotted against relative HORMAD1 expression in each sample.

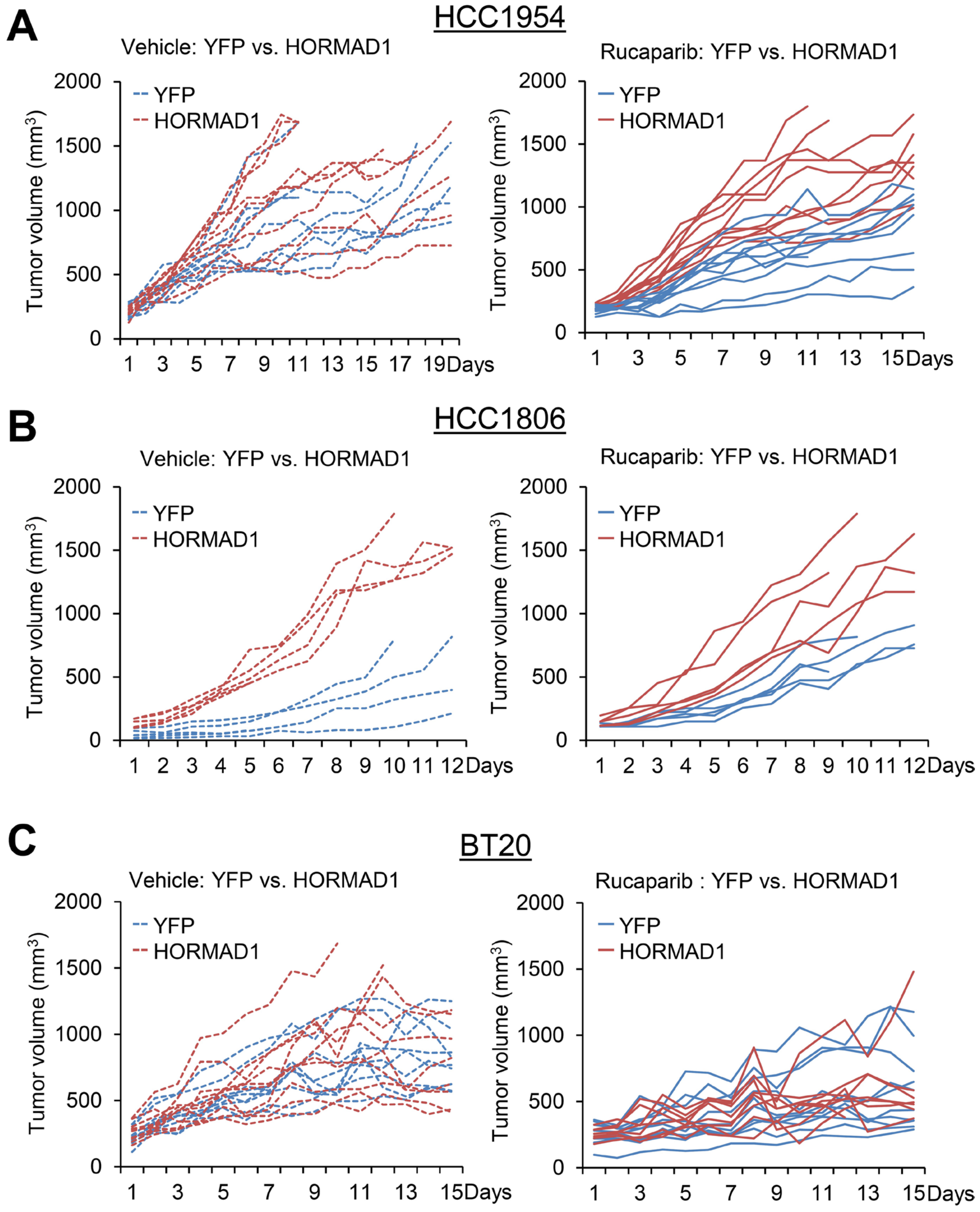

Supplementary Figure 7: Individual tumor growth curves for three HORMAD1-low basal-like breast cancer xenograft models. HCC1954 (**A**), HCC1806 (**B**), and BT20 (**C**). Annotation: YFP (blue): BLBC cell line stably overexpressing ORF of yellow fluorescent protein. HORMAD1 (red): BLBC cell lines stably overexpressing ORF of HORMAD1. All tumors are treated with either vehicle control (left panels) or Rucaparib (right panels).

Supplementary Table 1: A CpG island around the HORMAD1 transcription start site was amplified and the methylation status of 11 CpG sites within this region were examined by pyrosequecing. See Supplementary_Table_1

Supplementary Table 2: Clinical information as well as the HORMAD1 expression level and transcription start site CpG DNA methylation level of the TNBC tissues collected from Baylor tumor bank. See Supplementary_Table_2

Supplementary Table 3: Oncomine Roth Normal Tissue (grouped by normal tissue type). See Supplementary_Table_3
