# Supplementary material for: Epigenetic activation of HORMAD1 in basal-like breast cancer: role in Rucaparib sensitivity
Source: Oncotarget. 2018 Jul 10;9(53):30115–27. doi: 10.18632/oncotarget.25728 (PMC6059019; doi:10.18632/oncotarget.25728)
Supplement: Supplementary file 2 [file oncotarget-09-30115-s002.docx]

**Supplementary Table** **1:** A CpG island around the HORMAD1 transcription start site was amplified and the methylation status of 11 CpG sites within this region were examined by pyrosequecing. The relative expression level of HORMAD1 was determined by QPCR. The primers used for pyrosequecing and qPCR are stored in this file.

| **HORMAD1 pyrosequencing primer sequences** |
| --- |
| **PCR_F primer** |
| Hormad1_IL1_F1: GAAAATTAAAAGTTAGAAAAATAATTAGAA |
| Hormad1_IL1_F1_Biotin: GAAAATTAAAAGTTAGAAAAATAATTAGAA |
| Hormad1-F3: GATTAGGGGTTAAAAAGTTATT |
| GATTAGGGGTTAAAAAGTTATT |
| GATTAGGGGTTAAAAAGTTATT |
| Hormad1-F3_Biotin: GATTAGGGGTTAAAAAGTTATT |
| **PCR_R primer** |
| Hormad1_IL1_R4_Biotin: ACAATAACTTTTTAACCCCT |
| Hormad1_IL1_R4: ACAATAACTTTTTAACCCCT |
| Hormad1-R3 Biotin: CCATCTCAAAAACCTCTATTA |
| Hormad1-R3 Biotin: CCATCTCAAAAACCTCTATTA |
| Hormad1-R3 Biotin: CCATCTCAAAAACCTCTATTA |
| Hormad1_IL1_R2: CCTTAAAAAACTTAAAAATAAAAAAAA |
| **Sequencing primer** |
| Hormad1_IL1_R4_Biotin: GTGTTTGAGGTTTTTAGTG |
| Hormad1_IL1_R4: CACTAAAAACCTCAAACA |
| Hormad1-R3 Biotin: GGGGTTAAAAAGTTATTG |
| Hormad1-R3 Biotin: GGTGATYGTTGAAGGAAAG |
| Hormad1-R3 Biotin: GTGTAGTTTTTTTGGTTTT |
| Hormad1_IL1_R2: AATATATACACACTAATATATATTACATTA |
| **Sequence readout** |
| Hormad1_IL1_R4_Biotin: YGTTTTGYGTTGYGTTTGGYGGGAAAGGTGATTTYGA |
| Hormad1_IL1_R4: CRAAAAACCTCAACRCCACRTC |
| Hormad1-R3 Biotin: TYGYGTTTTAGYGGGTGATYGTTG |
| Hormad1-R3 Biotin: YGTATGYGYGTYGGGTATAGYGYGTGTAGTTTYGTG |
| Hormad1-R3 Biotin: YGGYGTTYGTTTTTTAGAYGTAA |
| Hormad1_IL1_R2: CRTCTAAAAAACRAACRCCRAAAACC |
| **HORMAD1 RT-PCR primers sequence** |
|  |
| **HORMAD1 primer set 1** |
| Forward primer: GCCCAGTTGCAGAGGACTC |
| Reverse primer: TCTTGTTCCATAAGCGCATTCT |
|  |
| **HORMAD1 primer set 2** |
| Forward primer: TGGCAAATGGAAATCAACCAGT |
| Reverse primer: TGCAAGCCTGCAGAACAAAA |
