# Supplementary material for: Epigenetic activation of HORMAD1 in basal-like breast cancer: role in Rucaparib sensitivity
Source: Oncotarget. 2018 Jul 10;9(53):30115–27. doi: 10.18632/oncotarget.25728 (PMC6059019; doi:10.18632/oncotarget.25728)
Supplement: Supplementary file 3 [file oncotarget-09-30115-s003.docx]

**Supplementary Table 2: Clinical information as well as the HORMAD1 expression level and transcription start site CpG DNA methylation level of the TNBC tissues collected from Baylor tumor bank.**

| **Sample_ID** | **AGE EXCISION** | **GENDER** | **RACE** | **HORMAD1 Relative mRNA level** | **HORMAD1 TSS CpG island DNA methylation %** | **MENOPAUSE STATUS** | **EXCISION YEAR** | **GRADE** | **BIOSAMPLE_CON_DIAG** | **HIST** | **AJCC STAGE** | **SIZE** | **POS NODE** | **METS** | **STAGE** | **ER** | **PR** | **HER** |
| --- | --- | --- | --- | --- | --- | --- | --- | --- | --- | --- | --- | --- | --- | --- | --- | --- | --- | --- |
| BT01 | 55 | Female | Caucasian | 3.203 | 95.655 | Post-Menopausal | 2006 | III | Infiltrating Ductal Carcinoma | IDC | T2N1M0 | >2cm, <=5cm | 1-3 Nodes(+) | No Mets Dz | Stage IIB | - | - | - |
| BT02 | 78 | Female | Caucasian | 0.075 | 95.475 | Post-menopausal | 2006 |  | Infiltrating Ductal Carcinoma | IDC | T2NXM0 | >2cm, <=5cm | unknown | No Mets Dz |  | - | - | - |
| BT03 | 69 | Female | Caucasian | 2.469 | 96.208 | Post-menopausal | 2006 | III | Infiltrating Ductal Carcinoma | IDC | T2N1M0 | >2cm, <=5cm | 1-3 Nodes(+) | No Mets Dz | Stage IIB | - | - | - |
| BT04 | 68 | Female | Caucasian | 4.086 | 90.938 | Post-menopausal | 2006 | III | Infiltrating Ductal Carcinoma | IDC | T2NXM0 | >2cm, <=5cm | unknown | No Mets Dz |  | - | - | - |
| BT05 | 55 | Female | Caucasian | #N/A | #N/A | Menopausal | 2007 |  | Infiltrating Ductal Carcinoma | IDC | T2N2MX | >2cm, <=5cm | 4-9 Nodes(+) | Unknown | Stage IIIA | - | - | - |
| BT06 | 74 | Female |  | #N/A | #N/A |  | 2006 |  | Infiltrating Ductal Carcinoma | IDC | T2NXM0 | >2cm, <=5cm | unknown | No Mets Dz |  | - | - | - |
| BT07 | 52 | Female |  | 2.392 | 29.344 | Post-menopausal | 2005 | III | Infiltrating Ductal Carcinoma | IDC | T3N2M0 | >5cm | 4-9 Nodes(+) | No Mets Dz | Stage IIIA | - | - | - |
| BT08 | 73 | Female | Caucasian | 6.892 | 37.566 | Pre-menopausal | 2005 | II | Infiltrating Ductal Carcinoma | IDC | T2N1M0 | >2cm, <=5cm | 1-3 Nodes(+) | No Mets Dz | Stage IIB | - | - | - |
| BT09 | 63 | Female | Asian | 1.911 | 76.874 |  | 2005 | III | Infiltrating Ductal Carcinoma | IDC | T2N0M0 | >2cm, <=5cm | Node (-) | No Mets Dz | Stage IIA | - | - | - |
| BT10 | 44 | Female | Caucasian | 2.015 | 95.635 |  | 2006 | III | Infiltrating Ductal Carcinoma | IDC | T2N0M0 | >2cm, <=5cm | Node (-) | No Mets Dz | Stage IIA | - | - | - |
| BT11 | 58 | Female | Caucasian | #N/A | #N/A |  | 2006 | II | Infiltrating Ductal Carcinoma | IDC | T2N1M0 | >2cm, <=5cm | 1-3 Nodes(+) | No Mets Dz | Stage IIB | - | - | - |
| BT12 | 52 | Female | Caucasian | 1.806 | 95.870 |  | 2007 | III | Infiltrating Ductal Carcinoma | IDC | T2N0M0 | >2cm, <=5cm | Node (-) | No Mets Dz | Stage IIA | - | - | - |
| BT13 | 65 | Female | Caucasian | 0.018 | 95.894 | Post-menopausal | 2007 | III | Infiltrating Ductal Carcinoma | IDC | TisN0M0 | unknown: CIS | Node (-) | No Mets Dz | AJCCStage IIA00Stage IIA Stage 0 | - | - | - |
| BT14 | 0 | Female | Caucasian | #N/A | #N/A | Pre-menopausal |  | II | Infiltrating Ductal Carcinoma | IDC | T2N0M0 | >2cm, <=5cm | Node (-) | No Mets Dz | Stage IIA | - | - | - |
| BT15 | 52 | Female | Caucasian | 0.008 | 95.033 | Menopausal | 2007 | III | Infiltrating Ductal Carcinoma | IDC | T1cN2M0 | <=2cm | 4-9 Nodes(+) | No Mets Dz | Stage IIIA | - | - | - |
| BT16 | 57 | Female | Caucasian | 0.046 | 94.020 | Menopausal | 2007 |  | Infiltrating Lobular Carcinoma | ILC | T1cN0M0 | <=2cm | Node (-) | No Mets Dz | Stage I | - | - | - |
| BT17 | 69 | Female | Caucasian | 0.015 | 95.866 | Post-Menopausal | 2007 | III | Infiltrating Ductal Carcinoma | IDC | T2NXM0 | >2cm, <=5cm | unknown | No Mets Dz |  | - | - | - |
| BT18 | 28 | Female | Caucasian | 4.909 | 96.234 | Pre-menopausal | 2007 | II | Infiltrating Ductal Carcinoma | IDC | T2N0MX | >2cm, <=5cm | Node (-) | Unknown | Stage IIA | - | - | - |
| BT19 | 60 | Female | Caucasian | 0.021 | 96.713 | Post-menopausal | 2007 | II | Infiltrating Ductal Carcinoma | IDC | T3N0MX | >5cm | Node (-) | Unknown | Stage IIB | - | - | - |
| BT20 | 58 | Female | Caucasian | #N/A | #N/A |  | 2007 | III | Infiltrating Ductal Carcinoma | IDC | T1cN0MX | <=2cm | Node (-) | Unknown | Stage I | - | - | - |
| BT21 | 54 | Female | Caucasian | 8.595 | 97.030 | Menopausal | 2007 | III | Adenocarcinoma | IDC | T2NXM0 | >2cm, <=5cm | unknown | No Mets Dz |  | - | - | - |
| BT22 | 65 | Female | Caucasian | 0.100 | 96.573 |  | 2007 | II | Infiltrating Ductal Carcinoma | IDC | T1N2M0 | <=2cm | 4-9 Nodes(+) | No Mets Dz | Stage IIIA | - | - | - |
| BT23 | 53 | Female | Caucasian | 0.013 | 95.703 |  | 2007 | II | Infiltrating Ductal Carcinoma | IDC | T2N2M0 | >2cm, <=5cm | 4-9 Nodes(+) | No Mets Dz | Stage IIIA | - | - | - |
| BT24 | 34 | Female | Caucasian | 0.007 | 93.021 | Pre-Menopausal | 2007 | III | Infiltrating Ductal Carcinoma | IDC | T2N1M0 | >2cm, <=5cm | 1-3 Nodes(+) | No Mets Dz | Stage IIB | - | - | - |
| BT25 | 22 | Female | Caucasian | 3.876 | 34.374 | Pre-Menopausal | 2007 | III | Infiltrating Ductal Carcinoma | IDC | T3N0M0 | >5cm | Node (-) | No Mets Dz | Stage IIB | - | - | - |
| BT26 | 77 | Female | Asian/Pacific Islander | 0.030 | 96.652 |  | 2007 | II | Infiltrating Ductal Carcinoma | IDC | T3N0M0 | >5cm | Node (-) | No Mets Dz | Stage IIB | - | - | - |
| BT27 | 42 | Female | Caucasian | 1.493 | 96.923 | Pre-menopausal | 2008 | III | Infiltrating Ductal Carcinoma | IDC | T2NXMX | >2cm, <=5cm | unknown | Unknown |  | - | - | - |
| BT28 | 45 | Female | Caucasian | #N/A | #N/A | Pre-menopausal | 2008 | II | Infiltrating Ductal Carcinoma | IDC | T1cN0M0 | <=2cm | Node (-) | No Mets Dz | Stage I | - | - | - |
| BT29 | 58 | Female | Caucasian | 0.576 | 78.047 | Post-menopausal | 2007 | III | Infiltrating Ductal Carcinoma | IDC | T1cN0M0 | <=2cm | Node (-) | No Mets Dz | Stage I | - | - | - |
| BT30 | 72 | Female | Caucasian | #N/A | #N/A | Post-menopausal | 2008 | II | Infiltrating Ductal Carcinoma | IDC | T3N3M0 | >5cm | >=10 Nodes(+) | No Mets Dz | Stage IIIC | - | - | - |
| BT31 | 57 | Female | Caucasian | #N/A | #N/A | Post-menopausal | 2008 | III | Infiltrating Ductal Carcinoma | IDC |  | unknown |  |  |  | - | - | - |
| BT32 | 72 | Female | Caucasian | 3.854 | 61.280 | Post-menopausal | 2004 |  | Medullary Carcinoma | Other Breast Cancer | T2N0M0 | >2cm, <=5cm | Node (-) | No Mets Dz | Stage IIA | - | - | - |
| BT33 | 39 | Female | Caucasian | 0.032 | 96.860 | Pre-menopausal | 2004 | III | Infiltrating Ductal Carcinoma | IDC | T1N1MX | <=2cm | 1-3 Nodes(+) | Unknown | Stage IIA | - | - | - |
| BT34 | 53 | Female | Caucasian | 0.028 | 97.382 | Pre-menopausal | 2005 | II | Infiltrating Ductal Carcinoma | IDC | T1cNXM0 | <=2cm | unknown | No Mets Dz |  | - | - | - |
| BT35 | 66 | Female | Caucasian | 0.151 | 96.544 | Post-menopausal | 2005 | III | Infiltrating Ductal Carcinoma | IDC | T1N0M0 | <=2cm | Node (-) | No Mets Dz | Stage I | - | - | - |
| BT36 | 58 | Female | Asian | 0.023 | 93.579 | Post-menopausal | 2002 | III | Infiltrating Ductal Carcinoma | IDC | T2N0M0 | >2cm, <=5cm | Node (-) | No Mets Dz | Stage IIA | - | - | - |
| BT37 | 54 | Female | Caucasian | #N/A | #N/A | Pre-menopausal | 2005 | III | Infiltrating Ductal Carcinoma | IDC | T3N2M0 | >5cm | 4-9 Nodes(+) | No Mets Dz | Stage IIIA | - | - | - |
| BT38 | 55 | Female | Caucasian | #N/A | #N/A | Post-menopausal | 2005 | III | Infiltrating Ductal Carcinoma | IDC | T2N1M0 | >2cm, <=5cm | 1-3 Nodes(+) | No Mets Dz | Stage IIB | - | - | - |
| BT39 | 59 | Female | Caucasian | 0.137 | 96.470 | Post-menopausal | 2005 | II | Infiltrating Ductal Carcinoma | IDC | T2N1M0 | >2cm, <=5cm | 1-3 Nodes(+) | No Mets Dz | Stage IIB | - | - | - |
| BT40 | 48 | Female | Caucasian | 0.006 | 96.279 | Menopausal | 2005 | II | Ductal Carcinoma In Situ (DCIS) | DCIS/LCIS | T3N2M0 | unknown: CIS | 4-9 Nodes(+) | No Mets Dz | Stage IIIA | - | - | - |
| BT41 | 51 | Female | Caucasian | 0.051 | 94.414 | Menopausal | 2005 | II | Ductal Carcinoma In Situ (DCIS) | DCIS/LCIS | T1cN0M0 | unknown: CIS | Node (-) | No Mets Dz | Stage I | - | - | - |
| BT42 | 56 | Female | Caucasian | 1.000 | 95.925 | Pre-Menopausal | 2005 |  | Infiltrating Ductal Carcinoma | IDC | T1bN0M0 | <=2cm | Node (-) | No Mets Dz | Stage I | - | - | - |
| BT43 | 59 | Female | Caucasian | 0.979 | 97.697 | Post-menopausal | 2005 | II | Infiltrating Ductal Carcinoma | IDC | T4N0M0 | any size w/ direct extension | Node (-) | No Mets Dz | Stage IIIB | - | - | - |
| BT44 | 68 | Female | Caucasian | #N/A | #N/A | Post-menopausal | 2005 | III | Infiltrating Ductal Carcinoma | IDC | T4aN1aM0 | any size w/ direct extension | 1-3 Nodes(+) | No Mets Dz | Stage IIIB | - | - | - |
| BT45 | 58 | Female | Caucasian | 0.000 | 96.166 | Post-menopausal | 2006 | III | Infiltrating Ductal Carcinoma | IDC | T2N2M0 | >2cm, <=5cm | 4-9 Nodes(+) | No Mets Dz | Stage IIIA | - | - | - |
| BT46 | 59 | Female | Caucasian | 4.095 | 96.013 | Post-menopausal | 2003 |  | Infiltrating Ductal Carcinoma | IDC | T1cN0M0 | <=2cm | Node (-) | No Mets Dz | Stage I | - | - | - |
| BT47 | 53 | Female | Caucasian | 0.692 | 96.044 | Pre-menopausal | 2006 | II | Infiltrating Ductal Carcinoma | IDC | T2N0MX | >2cm, <=5cm | Node (-) | Unknown | Stage IIA | - | - | - |
| BT48 | 52 | Female | Caucasian | 0.003 | 95.622 | Post-menopausal | 2006 | II | Infiltrating Ductal Carcinoma | IDC | T4N2MX | any size w/ direct extension | 4-9 Nodes(+) | Unknown | Stage IIIB | - | - | - |
| BT49 | 49 | Female | Caucasian | 10.914 | 25.662 | Menopausal | 2006 |  | Infiltrating Ductal Carcinoma | IDC | T1NXM0 | <=2cm | unknown | No Mets Dz |  | - | - | - |
| BT50 | 0 | Female | Caucasian | 0.177 | 95.118 |  | 2006 | III | Infiltrating Ductal Carcinoma | IDC | T1N0M0 | <=2cm | Node (-) | No Mets Dz | Stage I | - | - | - |
| BT51 | 36 | Female | Asian/Pacific Islander | 13.568 | 68.609 | Pre-Menopausal | 2007 |  | Infiltrating Ductal Carcinoma | IDC | T2NXMX | >2cm, <=5cm | unknown | Unknown |  | - | - | - |
| BT52 | 64 | Female | Caucasian | 6.001 | 96.652 |  | 2005 | I | Infiltrating Ductal Carcinoma | IDC | T1N3M0 | <=2cm | >=10 Nodes(+) | No Mets Dz | Stage IIIC | - | - | - |
| BT53 | 58 | Female | Caucasian | 3.480 | 80.293 | Menopausal | 2006 | III | Infiltrating Ductal Carcinoma | IDC | T2N1MX | >2cm, <=5cm | 1-3 Nodes(+) | Unknown | Stage IIB | - | - | - |
| BT54 | 50 | Female | Caucasian | 1.895 | 86.290 | Post-Menopausal | 2007 | III | Infiltrating Ductal Carcinoma | IDC | T2N0M0 | >2cm, <=5cm | Node (-) | No Mets Dz | Stage IIA | - | - | - |
| BT55 | 53 | Female | African-American | 1.108 | 92.286 | Post-menopausal | 2004 |  | Infiltrating Ductal Carcinoma | IDC |  | unknown |  |  |  | - | - | unknown |
| BT56 | 69 | Female | Caucasian | 0.030 | 93.729 | Post-menopausal | 2006 | III | Infiltrating Ductal Carcinoma | Other Breast Cancer | T1cN0M0 | <=2cm | Node (-) | No Mets Dz | Stage I | - | - | unknown |
| BT57 | 45 | Female | African American | 0.023 | 95.658 | Pre-menopausal | 2006 | III | Infiltrating Ductal Carcinoma | IDC | T2N1MX | >2cm, <=5cm | 1-3 Nodes(+) | Unknown | Stage IIB | - | - | unknown |
