# Supplementary material for: Epigenetic activation of HORMAD1 in basal-like breast cancer: role in Rucaparib sensitivity
Source: Oncotarget. 2018 Jul 10;9(53):30115–27. doi: 10.18632/oncotarget.25728 (PMC6059019; doi:10.18632/oncotarget.25728)
Supplement: Supplementary file 4 [file oncotarget-09-30115-s004.docx]

**Supplementary Table** **3: Oncomine Roth Normal Tissue (grouped by normal tissue type)**

**Legend**

1. Accumbens Nucleus (14)

2. Adipose Tissue (3)

3. Adrenal Cortex (4)

4. Amygdala (8)

5. Aorta (4)

6. Bone Marrow (5)

7. Breast (2)

8. Bronchus (3)

9. Cardiac Atrium (4)

10. Cardiac Ventricle (3)

11. Caudate Nucleus (4)

12. Cecum (3)

13. Cerebellum (10)

14. Cerebral Cortex (9)

15. Cervix Uteri (5)

16. Colon (1)

17. Coronary Artery (3)

18. Corpus Callosum (9)

19. Deltoid (6)

20. Dorsal Root Ganglion (8)

21. Duodenum (1)

22. Endometrium (18)

23. Esophagus (4)

24. Fallopian Tube (3)

25. Fetal Brain (1)

26. Fetal Liver (1)

27. Frontal Lobe (12)

28. Fundus of the Stomach (4)

29. Gastric Cardia (3)

30. Globus Pallidus (6)

31. Heart (1)

32. Hippocampus (8)

33. Hypothalamus (8)

34. Jejunum (3)

35. Kidney (1)

36. Liver (4)

37. Lung (3)

38. Lymph Node (4)

39. Mammary Gland (3)

40. Medulla Oblongata (9)

41. Mesencephalon (9)

42. Myometrium (27)

43. Nipple (4)

44. Nodose Ganglion (8)

45. Occipital Lobe (8)

46. Omentum (4)

47. Oral Mucosa (4)

48. Ovary (5)

49. Pancreas (1)

50. Parietal Lobe (8)

51. Pars Compacta (5)

52. Pars Reticulata (4)

53. Penis (6)

54. Pericardium (1)

55. Peritoneum (1)

56. Pharyngeal Mucosa (4)

57. Pituitary Gland (6)

58. Placenta (1)

59. Pons Varolii (1)

60. Prostate Gland (13)

61. Putamen (13)

62. Pylorus (4)

63. Quadriceps Muscle of the Thigh (1)

64. Renal Cortex (4)

65. Renal Medulla (4)

66. Salivary Gland (5)

67. Saphenous Vein (3)

68. Skeletal Muscle Tissue (4)

69. Skin (7)

70. Small Intestine (2)

71. Spinal Cord (9)

72. Spleen (5)

73. Stomach (1)

74. Subcutaneous Adipose Tissue (3)

75. Substantia Nigra (7)

76. Subthalamic Nucleus (9)

77. Superior Vestibular Nucleus (7)

78. Synovial Membrane (9)

79. Temporal Lobe (8)

80. Testis (6)

81. Thalamus (8)

82. Thymus Gland (7)

83. Tongue (10)

84. Tonsil (3)

85. Trachea (4)

86. Trigeminal Ganglion (8)

87. Umbilical Vein (2)

88. Urethra (5)

89. Uterus (1)

90. Vagina (4)

91. Vena Cava (1)

92. Ventral Tegmentum (8)

93. Ventrolateral Nucleus of the Thalamus (2)

94. Vermis (1)

95. Vulva (4)
